# The effects of 12 weeks of beta-hydroxy-beta-methylbutyrate free acid supplementation on muscle mass, strength, and power in resistance-trained individuals: a randomized, double-blind, placebo-controlled study

**DOI:** 10.1007/s00421-014-2854-5

**Published:** 2014-03-06

**Authors:** Jacob M. Wilson, Ryan P. Lowery, Jordan M. Joy, J. C. Andersen, Stephanie M. C. Wilson, Jeffrey R. Stout, Nevine Duncan, John C. Fuller, Shawn M. Baier, Marshall A. Naimo, John Rathmacher

**Affiliations:** 1 Department of Health Sciences and Human Performance, The University of Tampa, Tampa, FL 33606 USA; 2 Department of Nutrition, IMG Academy, Bradenton, FL USA; 3 Institute of Exercise Physiology and Wellness, University of Central Florida, Orlando, FL USA; 4 Metabolic Technologies Inc, Iowa State University Research Park, Ames, IA USA; 5 Department of Animal Science, Iowa State University, Ames, IA USA

**Keywords:** Leucine metabolite, Resistance training, Overreaching, Recovery, Sports supplements

## Abstract

**Introduction:**

Studies utilizing beta-hydroxy-beta-methylbutyrate (HMB) supplementation in trained populations are limited. No long-term studies utilizing HMB free acid (HMB-FA) have been conducted. Therefore, we investigated the effects of 12 weeks of HMB-FA supplementation on skeletal muscle hypertrophy, body composition, strength, and power in trained individuals. We also determined the effects of HMB-FA on muscle damage and performance during an overreaching cycle.

**Methods:**

A three-phase double-blind, placebo- and diet-controlled randomized intervention study was conducted. Phase 1 was an 8-week-periodized resistance-training program; Phase 2 was a 2-week overreaching cycle; and Phase 3 was a 2-week taper. Muscle mass, strength, and power were examined at weeks 0, 4, 8, and 12 to assess the chronic effects of HMB-FA; and assessment of these, as well as cortisol, testosterone, and creatine kinase (CK) was performed at weeks 9 and 10 of the overreaching cycle.

**Results:**

HMB-FA resulted in increased total strength (bench press, squat, and deadlift combined) over the 12-week training (77.1 ± 18.4 vs. 25.3 ± 22.0 kg, *p* < 0.001); a greater increase in vertical jump power (991 ± 168 vs. 630 ± 167 W, *p* < 0.001); and increased lean body mass gain (7.4 ± 4.2 vs. 2.1 ± 6.1 kg, *p* < 0.001) in HMB-FA- and placebo-supplemented groups, respectively. During the overreaching cycle, HMB-FA attenuated increases in CK (−6 ± 91 vs. 277 ± 229 IU/l, *p* < 0.001) and cortisol (−0.2 ± 2.9 vs. 4.5 ± 1.7 μg/dl, *p* < 0.003) in the HMB-FA- and placebo-supplemented groups, respectively.

**Conclusions:**

These results suggest that HMB-FA enhances hypertrophy, strength, and power following chronic resistance training, and prevents decrements in performance following the overreaching.

**Electronic supplementary material:**

The online version of this article (doi:10.1007/s00421-014-2854-5) contains supplementary material, which is available to authorized users.

## Introduction

Nearly two decades ago, Nissen et al. (1996) became the first to demonstrate that supplementation of the leucine metabolite, beta-hydroxy-beta-methylbutyrate (HMB), combined with resistance training improved protein balance and augmented gains in lean body mass (LBM) and strength. Since that time the most robust effects of HMB have been demonstrated in untrained individuals, who have experienced increased LBM and strength in as little as 3 weeks following supplementation (Jowko et al. 2001; Nissen et al. 1996). Presently, HMB is thought to operate via enhanced recovery of damaged skeletal muscle tissue (Wilson et al. 2008). Thus, these findings are not surprising as research clearly indicates that the initial weeks of training result in the highest magnitude of damage in an untrained population (Clarkson and Hubal 2002; McHugh et al. 1999). However, novices become resistant to damage during non-periodized training programs and their adaptations diminish (Turner 2011). Therefore, it is likely that the most important independent variables in any study examining HMB supplementation in athletes may be the intensity and variability of the training protocol.

To date, the overwhelming majority of studies in trained individuals have been non-periodized and unsupervised (Kreider et al. 1999; Thomson et al. 2009). The first study conducted in trained individuals supplementing with HMB found no differences between conditions. However, the investigators of this study did not monitor the training of their participants and instructed them to maintain the same training routine that they had prior to the start of the study (Kreider et al. 1999). Currently, only one study has included a high-intensity, monitored, resistance-training protocol in highly trained athletes (Nissen et al. 1996). However, this study lacked a high degree of variation, and was only 7 weeks in duration. Thus, there is a clear need for more long-term studies in resistance-trained populations, subjected to periodized, high-intensity resistance-training protocols. Moreover, it is essential to utilize direct measures of skeletal muscle hypertrophy and to monitor each resistance-training session to limit potential confounding variables that may have an effect on a true outcome.

Recently, HMB in a free acid form (HMB-FA) has been developed with improved bioavailability (Fuller et al. 2011). Initial studies have shown that this form of HMB supplementation results in approximately double the plasma levels of HMB in about one-quarter the time after administration when compared with the presently available form calcium HMB (HMB-Ca). In addition, this study showed that the HMB-FA had a 25 % greater clearance by the body, indicating improved utilization. Further, we have found that HMB-FA given 30 min prior to an acute bout of high-volume resistance training was able to attenuate indices of muscle damage and improve perceived recovery in resistance-trained athletes (Wilson et al. 2013b). More recently, acute ingestion of 3.4 g of HMB-FA has been demonstrated to increase skeletal muscle protein synthesis and decrease protein breakdown by +70 and −56 %, respectively (Wilkinson et al. 2013). To date, however, the majority of studies have been conducted using HMB-Ca (Wilson et al. 2008; 2013a).

Therefore, the primary purpose of this study was to investigate the effects of 12 weeks of HMB-FA supplementation in resistance-trained individuals during a monitored periodized resistance-training program on skeletal muscle hypertrophy, body composition, strength, and power relative to a placebo-matched control group. The secondary purpose of our investigation was to determine if HMB-FA was able to prevent the typical decay seen in performance following an overreaching cycle performed in the 9th and 10th weeks of the study.

## Methods

### Overview

The current study was a randomized, double-blind, placebo- and diet-controlled experiment consisting of a 12-week-periodized resistance-training program. The training protocol was divided into three phases and consisted of a non-linear-periodized resistance-training program for the first 8 weeks, followed by a 2-week overreaching cycle, and finally a 2-week taper of the training volume (Tables S1–S3). Muscle mass, body composition, strength, power, resting plasma testosterone, cortisol, and creatine kinase (CK) levels were examined collectively at the end of weeks 0, 4, 8, and 12 to assess the chronic effects of HMB; these measures were also assessed at the end of weeks 9 and 10, corresponding to the mid- and endpoints of the phase 2 overreaching cycle. The study was approved by the University of Tampa Institutional Review Board and registered with ClinicalTrials.gov (NCT01508338).

### Participants

Twenty-four resistance-trained males were tested and included in the study. Subjects were organized into quartile blocks based on their LBM and strength. Following this, each quartile was randomized to one of the treatment groups using computer-generated random numbers. After treatment assignment, the groups were assessed to confirm there were no differences between groups. Of those randomized to the treatments, three subjects dropped from the placebo group, two because of injury and one because of the time commitment, while one dropped from the HMB-FA group due to injury. All drop outs occurred during the first 4 weeks of the study. The remaining 20 subjects (21.6 ± 0.5 years of age) consisting of nine placebo (87.1 ± 4.8 kg; 180.9 cm) and 11 supplemented (83.1 ± 2.8 kg; 179.0 ± 2.1) males with an average squat, bench press, and deadlift of 1.7 ± 0.04, 1.3 ± 0.04, and 2.0 ± 0.05 times their bodyweight, respectively, completed the study. There were no significant differences for age, body weight, height, or BMI between the treatment groups at the start of the study. Participants were excluded if they were currently taking anti-inflammatory agents as well as any other performance-enhancing supplement, or if they smoked. The participants must not have taken any nutritional supplements for at least 3 months prior to the start of data collection. Each participant signed an informed consent approved by the University of Tampa Institutional Review Board before participating in the study in the Department of Health Sciences and Human Performance at the University of Tampa, Tampa, FL, USA.

### Strength, power, body composition, and skeletal muscle hypertrophy testing

Strength was assessed via a one-repetition maximum (1-RM) testing of the back squat, bench press, and deadlift. The intraclass correlation coefficient (ICC) for the test–retest of strength in the squat, bench press, and deadlift ranged from *r* = 0.956 to 0.982. Body composition [LBM, fat mass (FM), and total mass] was determined on a Lunar Prodigy dual X-ray absorptiometry (DXA) apparatus (software version, enCORE 2008, Madison, WI, USA). Tests for the DXA were performed at the same time of day in a fasted state and the ICC was *r* = 0.981. Skeletal muscle hypertrophy was assessed via changes in ultrasonography (GE Logiq e 2008, Wauwatosa, WI, USA) determined combined muscle thickness of the vastus lateralis (VL) and vastus intermedius (VI) muscles. The ICC for the test–retest of muscle thickness measurements was *r* = 0.975.

Peak power (PP) was assessed during maximal cycling (modified Wingate test) and jumping movements. During the cycling test, the volunteer was instructed to cycle against a predetermined resistance (7.5 % of body weight) as fast as possible for 10 s (Smith et al. 2001) on a cycle ergometer (Monark model 894e, Vansbro, Sweden). Wingate PP was recorded using Monark anaerobic test software (Version 1.0, Monark, Vansbro, Sweden). From completion of Wingate tests performed over several days, the ICC for Wingate PP was 0.966.

Measurements of PP for the vertical jump were also taken on a multicomponent AMTI force platform (Advanced Mechanical Technology, Watertown, MA USA) which interfaced with a personal computer at a sampling rate of 1,000 Hz (Lowery et al. 2012). Data acquisition software (LabVIEW, version 7.1; National Instruments Corporation, Austin, TX, USA) was used to calculate vertical jump PP. The ICC for VJ PP was 0.971.

### Supplementation, diet control, and exercise protocol

Prior to the study, participants were randomly assigned to receive either 3 g per day of HMB-FA or a placebo divided equally into three servings. Each serving was formulated with 1 g of HMB-FA. The first serving was given 30 min prior to exercise and the remaining two servings given with the mid-day and evening meals. On the non-training days, participants were instructed to consume one serving with each of three separate meals throughout the day. Blinding occurred via an outside researcher who sent an isocaloric supplement and placebo in identical looking and flavored packets containing either 3 g per day of HMB-FA (combined with food-grade orange flavors and sweeteners) or a placebo (corn syrup combined with food-grade orange flavors and sweeteners) divided equally into three servings daily. This researcher was not involved in direct data collection, or statistical analysis, and did not meet any of the subjects. For this reason, neither the researchers conducting the study, nor the subjects knew which participant was assigned to which group. Moreover, the code was not broken until after all of the data were entered into a computer spreadsheet, and sent to an outside researcher who was also blinded to the treatment groups. The supplementation was continued daily throughout the training and testing protocols. Two weeks prior to and throughout the study, participants were placed on a diet consisting of 25 % protein, 50 % carbohydrates, and 25 % fat by a registered dietician (M.S., RD, LD) who specialized in sports nutrition. The participants met as a group with the dietitian, and they were given individual meal plans at the beginning of the study. Diet counseling was continued throughout the duration of the study. Assessment of 3-day food records taken at the beginning, mid, and last week of the study revealed that diets consisted of 22 % protein, 45 % carbohydrates, and 33 % fat, with no differences between groups. Compliance of supplementation was assessed by having the participants hand their empty packets to a researcher at the beginning of each training day. Compliance was over 98 % for supplementation.

The purity of HMB-FA was determined by the manufacturer (TSI, Missoula, MT, USA) using high-pressure liquid chromatography to be 99.7 %. The primary impurities were acetate and water. Metabolic Technologies Inc. (MTI, Ames, IA, USA) independently assayed the HMB-FA for purity and confirmed these results. In addition, MTI assayed HMB-FA for dehydroepiandrosterone (DHEA) a contaminant which has been found in nutritional supplements using gas chromatography–mass spectrometry (Thuyne and Delbeke 2005). DHEA was not detected in the HMB-FA (<1 ng/g). In addition, a sample was sent to an independent laboratory (MVTL, New Ulm, MN, USA) for microbial and heavy metals testing. HMB-FA tested negative for *E. coli*, *Listeria*, *and Salmonella.* Copper, zinc, calcium, mercury, cadmium, and lead were less than the instrument’s detection limits and arsenic was detected at 37 PPB.

### Exercise protocols

The training was divided into three phases, with Phase 1 (Table S1) consisting of a daily undulating periodized resistance-training program 3 days per week during weeks 1 through 8. This protocol was modified from Kraemer et al. (2009). Phase 2 (Table S2) consisted of a 2-week overreaching cycle during weeks 9 and 10 wherein participants resistance trained 5 days per week and also performed an additional day of Wingate and power testing. Finally, phase 3 (Table S3) consisted of a tapered training volume for weeks 11 and 12. All training sessions were monitored and controlled by the researchers, and if a session was missed at a specific time of day it was made up within 24 h. Using this criterion, compliance was 100 % for all subjects who completed the study.

### Resting blood draws

All blood draws throughout the study were obtained via venipuncture after a 12 h fast by a trained phlebotomist. All blood draws were scheduled at the same time of day to negate confounding influences of diurnal hormonal variations. Whole blood was collected and transferred into appropriate tubes for obtaining serum and plasma and centrifuged at 1,500 g for 15 min at 4 °C. Resulting serum and plasma were then aliquoted and stored at −80 °C until subsequent analyses. A portion of the blood samples taken at weeks 0, 4, 8, and 12 were used for measurements (Any Lab Test Now^®^, Tampa, Fl, USA) of glucose, blood urea nitrogen, creatinine, eGFR, Na^+^, K^+^, Cl^−^, CO_2_, Ca^2+^, protein, albumin, globulin, albumin:globulin ratio, total bilirubin, alkaline phosphatase, aspartate aminotransferase, and alanine aminotransferase. A complete blood count was also performed on each blood sample. In addition, a urinalysis (urine specific gravity, pH, and Urobilinogen) was conducted on a sample of urine. The samples were submitted to an outside laboratory for analysis (Any Lab Test Now^®^, Tampa, FL, USA) at weeks 0, 4, 8, and 12.

### Biochemical analysis

Samples were thawed one time and analyzed in duplicate for each analyte. Serum total and free testosterone, cortisol, and C-reactive protein (CRP) were assayed via ELISA kits (Diagnostic Systems Laboratories, Webster, TX, USA). All hormones were measured in the same assay on the same day to avoid interassay variation. All samples were analyzed in duplicate. The intra-assay variance was calculated by coefficient of variation [(SD/mean) × 100]. Intra-assay variance was determined to be <3 % for all analytes. Serum CK was measured at 340 nm using colorimetric procedures (Diagnostics Chemicals, Oxford, CT, USA). As a measure of muscle protein degradation, the ratio of urinary 3-methylhistidine to creatinine (3MH:Cr) was calculated. The participants were instructed to consume a meat-free diet for 72 h prior to each measurement period at weeks 8, 9, and 10. Urine was collected for 24 h, uniformly mixed, sampled, and then stored at −80 °C until subsequent analyses. The 3MH was measured using a previously described GC/MS method (Rathmacher et al. 1992). Urinary creatinine (Cr) was measured using a colorimetric Jaffe’s reaction (Cayman Chemical, Ann Arbor, MI, USA). The 3MH:Cr ratio over the 24-h period was then calculated.

### Perceived recovery status scale

Perceived recovery status (PRS) scale was measured at weeks 0, 4, 8, 9, 10, and 12 to assess subjective recovery during the training phases. The PRS scale consists of values between 0 and 10, with 0–2 being very poorly recovered with anticipated declines in performance, 4–6 being low to moderately recovered with expected similar performance, and 8–10 representing high perceived recovery with expected increases in performance. The PRS scale has been demonstrated as a valid cognitive indicator of performance and fatigue (Laurent et al. 2011; Sikorski et al. 2013).

### Statistics

A one-way ANOVA model was used to analyze the baseline characteristic data using the Proc GLM procedure in SAS (SAS Institute, Cary, NC, USA). The primary outcome measure of this double-blind, placebo- and diet-controlled randomized intervention study was muscular strength and power. The secondary outcome measure was muscle hypertrophy. The main effect of treatment (Trt) was included in the model. Changes over the 12-week study were analyzed by repeated measures ANOVA using the Proc Mixed procedure in SAS with the initial week of the period, week 0, used as a covariate and the main effects were Time, Trt, and Trt × Time. In addition, the overreaching phase of the study was further assessed using a repeated measures ANOVA with Proc Mixed procedure in SAS with the week 8 time point used as a covariate and the main effects were Time, Trt, and Trt × Time. Least squares means procedure was then used to compare treatment means at each time point. The *n* size was based on a power analysis of LBM differences found by Kraemer et al. (2009). Statistical significance was determined at *p* ≤ 0.05. The results are presented as mean ± standard deviations.

## Results

### Muscle strength and power

HMB-FA supplementation resulted in a significant increase in strength gain compared with placebo supplementation for squat, bench press, deadlift, and total strength (Table 1). After 12 weeks of training, HMB-FA supplementation resulted in strength increases of 25 % for the squat, 12 % for the bench press, 16 % for the deadlift, and 18 % for total strength, which were significantly greater than the increases of 5 % for the squat, 3 % for the bench press, 9 % for the deadlift, and 6 % for total strength in the placebo group. Total strength increases over the 12-week study were 25.3 ± 22.0 kg in the placebo-supplemented participants, and 77.1 ± 18.4 kg in the HMB-FA-supplemented participants. Mean total strength also demonstrated that the HMB-FA supplementation group was significantly greater at 4, 8, and 12 weeks during the periodized resistance-training phases compared to the mean total strength in the placebo-supplemented group.

**Table 1:** Effect of beta-hydroxy-beta-methylbutyrate free acid (HMB-FA) supplementation on muscle strength and power in participants performing a 12-week resistance-training regimen

	Week of study				*p* value^a^
	0	4	8	12	
Total strength^b^ (kg)					
Placebo	426.7 ± 14.5	444.6 ± 14.5	457.8 ± 14.5	452.0 ± 14.5	
HMB-FA	426.7 ± 14.5	458.7 ± 14.5	477.6 ± 14.5	503.8 ± 14.5	0.0001
Squat (kg)					
Placebo	143.8 ± 5.2	150.4 ± 5.2	155.4 ± 5.2	151.1 ± 5.2	
HMB-FA	143.7 ± 5.2	154.9 ± 5.2	162.4 ± 5.2^#^	179.9 ± 5.2^#^	0.0001
Bench press (kg)					
Placebo	112.9 ± 6.6	116.4 ± 6.6	118.5 ± 6.6	116.7 ± 6.6	
HMB-FA	112.4 ± 6.6	120.8 ± 6.6	123.7 ± 6.6	125.2 ± 6.6^#^	0.02
Deadlift (kg)					
Placebo	170.4 ± 9.2	178.2 ± 9.2	184.3 ± 9.2	184.5 ± 9.2	
HMB-FA	170.3 ± 9.2	182.7 ± 9.2	191.2 ± 9.2	198.4 ± 9.2^#^	0.009
Wingate peak power (W)					
Placebo	879.1 ± 38.3	927.0 ± 38.3	987.2 ± 38.3	982.5 ± 38.3	
HMB-FA	879.7 ± 38.3	936.0 ± 38.3	980.7 ± 38.3	1,038.6 ± 38.3^#^	0.01
Vertical jump power (W)					
Placebo	5,224 ± 73	5,636 ± 73	5,839 ± 73	5,854 ± 73	
HMB-FA	5,219 ± 73	5,835 ± 73^#^	6,039 ± 73^#^	6,211 ± 73^#^	0.001

Adjusted least square mean ± SD for *n* = 11 HMB (3 g HMB-FA/d in three 1 g doses) and *n* = 9 placebo-supplemented participants

^a^Probability of treatment by time difference between the placebo and the HMB-FA treatments over the 12-week study. The mixed model ANOVA in SAS^®^ was used with the main effects of treatment, time and treatment by time, with the value for week 0 used as a covariate

^b^Total strength as the sum of the 1-RM in bench press, squat, and deadlift

^#^Significantly different than corresponding placebo, *t* test (*p* < 0.05)

Wingate PP increased more in the HMB-FA-supplemented participants than in the placebo-supplemented participants over the 12-week study. HMB-FA-supplemented participants increased Wingate PP by 18 % compared with a 12 % increase in placebo-supplemented participants (Table 1). The 12-week mean Wingate PP was also significantly greater in the HMB-FA group compared to the placebo group. A similar difference in vertical jump power was seen between the HMB-FA group and placebo group). Vertical jump power in the HMB-FA group increased 19 % after 12 weeks compared to the placebo group increase of 12 %. The mean vertical jump power was significantly improved with HMB-FA supplementation in comparison to the placebo at weeks 4, 8, and 12.

The increase in training volume, and decrease in recovery during overreaching phase, significantly decreased strength in the placebo group (Table 2). These decrements consisted of average decrements of −5.6 % for the squat, −4.8 % for the bench press, and −4.5 % for total strength compared to the HMB-FA group, whom had average decrements in strength of −0.6 % for the squat, −1.0 % for the bench press, and −0.4 % for total strength. While not significantly different over both weeks of the overreaching cycle, the decrease in deadlift strength in the placebo group after the first week of overreach was significantly greater than the decrease in the HMB-FA group after 1 week of the overreaching phase, −5.6 vs. −1.5 %, respectively. The total strength decrease during the 2-week overreaching phase was −20.2 ± 15.2 kg in the placebo group, while the HMB-FA group decrement was −2.0 ± 18.6 kg, which was significantly different between groups. In addition, mean total strength was significantly greater in the HMB-FA group at weeks 9 and 10 compared with placebo group (Table 2).

**Table 2:** Effect of beta-hydroxy-beta-methylbutyrate free acid (HMB-FA) supplementation on muscle strength and power during the overreaching phase, weeks 8, 9, and 10, of a 12-week resistance-training regimen

	Week of study			*p* value^a^
	8	9	10	
Total strength^b^ (kg)				
Placebo	467.8 ± 11.9	443.6 ± 11.9	447.6 ± 11.9	
HMB-FA	469.4 ± 11.9	464.5 ± 11.9^#^	467.3 ± 11.9^#^	0.01
Squat (kg)				
Placebo	159.2 ± 4.8	152.6 ± 4.8	150.6 ± 4.8	
HMB-FA	159.3 ± 4.8	158.3 ± 4.8^#^	162.6 ± 4.8^#^	0.0001
Bench press (kg)				
Placebo	121.4 ± 4.7	113.5 ± 4.7	115.6 ± 4.7	
HMB-FA	121.4 ± 4.7	120.3 ± 4.7^#^	120.1 ± 4.7^#^	0.05
Deadlift (kg)				
Placebo	187.2 ± 6.9	177.3 ± 6.9	181.4 ± 6.9	
HMB-FA	188.8 ± 6.9	185.9 ± 6.9	184.7 ± 6.9	0.26
Wingate peak power (W)				
Placebo	983.9 ± 38.8	917.5 ± 38.8	939.5 ± 38.8	
HMB-FA	977.6 ± 38.8	965.4 ± 38.8^#^	972.7 ± 38.8^#^	0.04
Vertical jump power (W)				
Placebo	5,949 ± 57.5	5,723 ± 57.5	5,656 ± 57.5	
HMB-FA	5,949 ± 57.5	5,867 ± 57.5^#^	5,870 ± 57.5^#^	0.0001

Adjusted least square mean ± SD for *n* = 11 HMB (3 g HMB-FA/d in three 1 g doses) and *n* = 9 placebo-supplemented participants

^a^Probability of treatment by time difference between the placebo and the HMB-FA treatments over weeks 8, 9, and 10, the overreaching portion of the 12-week study. The mixed model in SAS was used with the main effects of treatment, week and treatment by week, with the value for week 8 as a covariate

^b^Total strength as the sum of the 1-RM in bench press, squat, and deadlift

^#^Significantly different than corresponding placebo, *t* test (*p* < 0.05)

### Body composition and muscle measurement

Supplementation with HMB-FA resulted in a significant increase in body mass and LBM compared with placebo supplementation (Table 3) during the 12-week exercise training period. The HMB-FA group also experienced a significant decrease in body fat, −5.4 ± 1.6 kg, compared with the placebo group, −1.7 ± 2.7 kg (Table 3). Supplementation with HMB-FA resulted in significantly greater quadriceps thickness compared to the placebo group over the 12-week training period.

**Table 3:** Effect of beta-hydroxy-beta-methylbutyrate free acid (HMB-FA) supplementation on body composition and quadriceps depth in participants performing a 12-week resistance-training regimen

	Week of study				
	0	4	8	12	*p* value^a^
Weight (kg)					
Placebo	84.8 ± 0.9	85.7 ± 0.9	86.0 ± 0.9	85.1 ± 0.9	
HMB-FA	85.0 ± 0.9	85.8 ± 0.9	86.7 ± 0.9	86.9 ± 0.9^#^	0.003
DXA LBM^b^ (kg)					
Placebo	67.1 ± 1.1	68.0 ± 1.1	70.0 ± 1.1	69.2 ± 1.1	
HMB-FA	67.1 ± 1.1	70.1 ± 1.1^#^	72.2 ± 1.1^#^	74.5 ± 1.1^#^	0.001
DXA fat (kg)					
Placebo	17.6 ± 1.7	16.8 ± 1.7	16.0 ± 1.7	15.9 ± 1.7	
HMB-FA	17.9 ± 1.7	15.7 ± 1.7	14.4 ± 1.7^#^	12.5 ± 1.7^#^	0.0003
Quadriceps depth (mm)					
Placebo	50.2 ± 2.1	52.2 ± 2.1	52.5 ± 2.1	52.6 ± 2.1	
HMB-FA	50.2 ± 2.1	53.1 ± 2.1	55.60 ± 2.1^#^	57.4 ± 2.1^#^	0.0001

Adjusted least square mean ± SD for *n* = 11 HMB-FA-supplemented (3 g HMB-FA/d in three 1 g doses) and *n* = 9 placebo-supplemented participants

^a^Probability of treatment by time difference between the placebo and HMB-FA treatments over the 12-week study. The mixed model ANOVA in SAS was used with the main effects of treatment, time and treatment by time, with the value for week 0 used as a covariate of treatment

^b^ *DXA LBM* Dual X-Ray absorptiometry determined lean body mass

^#^Significantly different than corresponding placebo, *t* test (*p* < 0.05)

### Muscle damage, hormonal status, and performance recovery scale

Supplementation with HMB-FA diminished the rise in CK following training over the 12-week study compared with the placebo-supplemented group (Table 4). At 4 weeks, CK levels were significantly less in the HMB-FA group compared with the placebo group. While no significant treatment effects were observed for serum CRP levels, serum cortisol levels decreased in the HMB-FA group in comparison to an increase in the placebo group. There were no significant changes in either free or total testosterone during the study (data not shown). Supplementation with HMB-FA improved the participants’ perceived recovery from the previous bouts of exercise compared with the placebo-supplemented group which decreased in perceived recovery (Table 4). In addition, the mean PRS in the HMB-FA group was significantly higher than the mean PRS indicated by the placebo group at weeks 8 and 12.

**Table 4:** Effect of beta-hydroxy-beta-methylbutyrate free acid (HMB-FA) supplementation on serum creatine kinase (CK), cortisol, and perceived recovery score (PRS) in participants performing a 12-week resistance-training regimen

	Week of study				*p* value^a^
	0	4	8	12	
Creatine kinase (IU/l)					
Placebo	146 ± 62.3	388 ± 62.3	261 ± 62.3	195 ± 62.3	
HMB-FA	154 ± 62.3	275 ± 62.3^#^	251 ± 62.3	142 ± 62.3	0.01
Cortisol (μg/dl)					
Placebo	20.3 ± 2.6	19.3 ± 2.6	19.9 ± 2.6	21.2 ± 2.6	
HMB-FA	21.0 ± 2.6	20.3 ± 2.6	18.2 ± 2.6	16.9 ± 2.6^#^	0.004
PRS^b^					
Placebo	9.1 ± 0.8	7.1 ± 0.8	7.7 ± 0.8	7.6 ± 0.8	
HMB-FA	9.1 ± 0.8	7.6 ± 0.8	8.5 ± 0.8^#^	9.5 ± 0.8^#^	0.003

Adjusted least square mean ± SD for *n* = 11 HMB-FA (3 g HMB-FA/d in three 1 g doses) and *n* = 9 placebo-supplemented participants

^a^Probability of treatment by time difference between the placebo and the HMB-FA treatments over the 12-week study. The mixed model in SAS was used with the main effects of treatment, week and treatment by week, with the value for week 0 used as a covariate

^b^Perceived recovery score is rated on the participants feeling of recovery from the last workout on a scale of 0–10

^#^Significantly different than corresponding placebo, *t* test (*p* < 0.05)

The increase in training volume with the overreaching phase resulted in a significant (108 %) increase in serum CK in the placebo group over the 2 weeks of training during weeks 9 and 10, while CK in the HMB-FA did not change. Serum CK levels in the HMB-FA group were significantly lower after each week, 9 and 10, of the overreaching phase. A similar decrease was observed for serum LDH in the HMB-FA group (Data not shown). Measurement of the urinary 3MH:Cr ratio, an indicator of the rate of muscle protein degradation, during the 2-week overreaching period showed no significant effect of HMB-FA supplementation (Table 5).

**Table 5:** Effect of beta-hydroxy-beta-methylbutyrate free acid (HMB-FA) supplementation on serum creatine kinase (CK), urinary 3-methylhistidine:creatinine ratio (3MH:Cr), cortisol, and perceived recovery score (PRS) in participants during the overreaching phase, weeks 8, 9, and 10, of a 12-week resistance-training regimen

	Week of study			*p* value^a^
	8	9	10	
24 h 3MH:Cr (μmol:mg)				
Placebo	0.124 ± 0.012	0.132 ± 0.012	0.152 ± 0.012	
HMB-FA	0.124 ± 0.012	0.120 ± 0.012	0.141 ± 0.012	0.34
Creatine kinase (IU/l)				
Placebo	256 ± 119.8	494 ± 119.8	533 ± 119.8	
HMB-FA	256 ± 119.8	288 ± 119.8^#^	250 ± 119.8^#^	0.0001
Cortisol (μg/dl)				
Placebo	19.1 ± 1.9	21.9 ± 1.9	23.5 ± 1.9	
HMB-FA	18.9 ± 1.9	19.8 ± 1.9^#^	18.8 ± 1.9^#^	0.003
PRS^b^				
Placebo	7.9 ± 0.8	5.0 ± 0.8	4.6 ± 0.8	
HMB-FA	8.4 ± 0.7	7.8 ± 0.7^#^	7.6 ± 0.7^#^	0.001

Adjusted least square mean ± SD for *n* = 11 HMB-FA (3 g HMB-FA free acid/d in three 1 g doses) and *n* = 9 placebo-supplemented participants

^a^Probability of treatment by time difference between the placebo and the HMB-FA treatments over weeks 8, 9, and 10, the overreaching portion of the 12-week study. The mixed model in SAS was used with the main effects of treatment, week and treatment by week, with the value for week 8 as a covariate

^b^Perceived recovery score is rated on the participants feeling of recovery from the last workout on a scale of 0–10

^#^Significantly different than corresponding placebo, *t* test (*p* < 0.05)

### Safety studies

There were no differences in blood chemistry and hematology between the placebo- and HMB-FA-supplemented groups. In addition, no differences were observed in urinalysis values between the groups. Finally, there were no reported adverse effects for use of the supplement or placebo. The blood chemistry and hematology, and urine data are presented in supplementary Tables S4–S6.

## Discussion

The primary findings of this study were that individuals consuming HMB-FA over 12 weeks of a periodized resistance-training program obtained greater gains in skeletal muscle hypertrophy, LBM, strength, and power in comparison to the placebo-supplemented group. The HMB-FA group also demonstrated increased loss of body fat compared with the placebo group. Finally, HMB-FA prevented declines in strength and power during the overreaching phase, and HMB-FA blunted the increase in muscle damage and cortisol during this period of time.

### The effects of HMB-FA on skeletal muscle hypertrophy and changes in lean body mass

Our results indicated greater increases in LBM and muscle thickness in the HMB-FA group as compared to the placebo group (Table 3). These results agreed with Kraemer et al. (2009), who also reported large treatment effects following a 12-week-periodized program; however, this was in an untrained population. Previous research in trained populations has been inconsistent, with findings of both no effect (Kreider et al. 1999; Slater et al. 2001), or positive treatment effects on indices of muscle mass (Nissen et al. 1996; Thomson et al. 2009). There are a number of possible reasons for the equivocal results observed across multiple study formats.

The literature suggests that in order to see an effect on skeletal muscle hypertrophy, the degree of undulation (e.g., periodization) and the duration of a training program should increase proportionally to the training status of the individual (Monteiro et al. 2009; Turner 2011). Previous studies which used HMB in trained populations have lasted from 28 days (Kreider et al. 1999) to as long as 9 weeks in length (Thomson et al. 2009). In general, when using HMB as an intervention in trained individuals, little to no changes in hypertrophy have been found in studies lasting <6 weeks in duration (Hoffman et al. 2004; Kreider et al. 1999; Slater et al. 2001). It is conceivable that a duration of <6 weeks is not long enough to see significant changes in hypertrophy or LBM in experienced, resistance-trained individuals (Ahtiainen et al. 2003). Prior to our research, the longest duration study examining HMB supplementation in a trained population was conducted by Thomson and colleagues (2009). This study contained unsupervised, non-periodized training sessions, had moderate (84 %) compliance, and utilized highly variable outcome measures of hypertrophy (bioelectrical impedance) (Loenneke et al. 2012). However, despite these limitations, 9 weeks of HMB-Ca supplementation resulted in small, but significant increases in fat free mass (Thomson et al. 2009).

Our study in highly trained individuals utilized an intricate design aimed at recapitulating skeletal muscle stress and damage often seen in individuals with low training experience (Kraemer et al. 2009). As such, our repetition scheme, intensity, and rest period lengths varied from workout to workout, from week to week, and from phase 1 through phase 3 of the study (Tables 1, 2, 3). Measures of CK confirmed that our design was novel enough to recapitulate the skeletal muscle stress and damage often seen in individuals with low training experience. As such, participants in the HMB-FA group experienced similar gains as has been previously observed in untrained populations. Collectively, these findings suggest that HMB can augment increases in hypertrophy and LBM in both untrained and trained populations so long as the training stimulus is of great enough relative magnitude to cause significant and frequent perturbations to skeletal muscle. Though the mechanisms of action involving HMB-FA have not fully been elucidated, it is currently thought to work through improving protein turnover and skeletal muscle regeneration (Wilson et al. 2008; Zanchi et al. 2011). These explanations were generally indicated in our study results by the overall lower skeletal muscle disruption throughout each measure of the 12 weeks of high-intensity training.

### The effects of HMB-FA on skeletal muscle strength and power development

Strength and power are two of the most critical attributes underlying success in sport (Robbins and Docherty 2005; Wilson et al. 2012). These variables are intimately related and allow athletes to be successful in their respective sport (Cormie et al. 2011a, b). The collective results of the present study, as well as those from Kraemer et al. (2009), suggest that changes in strength and power following HMB supplementation are optimized within the context of a periodized as compared to a non-periodized training program (Thomson et al. 2009). Moreover, it is conceivable that the magnitude of strength and power adaptations resulting from HMB supplementation may be reflective of the measurement technique. For example, past research utilizing compound, sport-specific movements such as the squat, bench press, and vertical jump have found robust changes in strength and power following HMB supplementation (Kraemer et al. 2009; Nissen et al. 1996). In contrast, researchers have found small treatment effects when using non-specific, isolated movements such as the leg extension and preacher curl (Thomson et al. 2009). Given that HMB augments skeletal muscle mass, it is likely that its benefits are more fully realized when expressed under multi-joint, compound movements (e.g., squat, deadlift), which stress a greater total amount of the skeletal muscle system.

### The effects of HMB-FA on body fat

Recent evidence from the laboratory of Michael Zemel at the University of Tennessee (Bruckbauer et al. 2012) has demonstrated that HMB supplementation improves fatty acid oxidation, adenosine monophosphate kinase (AMPK), Sirt1, and Sirt3 activity in mouse-derived 3T3-L1 adipocytes and C2C12 muscle cells. To elaborate, the Sirt proteins (Silent information regulator transcripts) belong to a class of NAD^+^-dependent protein deacetylases involved in energy metabolism, which sense energy balance through changes in the NAD^+^/NADH ratio. Sirt proteins modify the acetylation level of histones and proteins (Verdin et al. 2010). AMPK is also a sensor of energy balance, but does so through changes in AMP/ATP ratios (Hardie 2003). Collectively, these proteins act to improve mitochondrial biogenesis, fat oxidation, energy metabolism, and the reactive oxygen defense system (Hardie 2003; Hardie et al. 2006; Verdin et al. 2010). Consequently, this recent evidence has shown that HMB supplementation increases mitochondria biogenesis and fat oxidation (Stancliffe and Zemel 2012). While our primary aim was not to investigate HMB’s effects on fat mass, we did find that those supplementing with HMB-FA lowered body fat relative to the placebo group. These findings agreed with Kraemer et al. (2009), who also found that participants lost more body fat following 12 weeks of HMB supplementation relative to a placebo-matched control.

### The effects of HMB-FA on overreaching

The primary cause of overreaching appears to be an imbalance between the training stimulus and recovery. Our results indicated that our overreaching cycle was able to decrease power, strength, and perceived recovery in the placebo, but not in the HMB-FA group. Moreover, HMB-FA was able to blunt the characteristic rise in CK, an indicator of skeletal muscle damage, and an elevation in the serum stress hormone cortisol following the overreaching cycle. Finally, following a 2-week taper in which the training volume was lowered, the placebo group regained their baseline performance, while the HMB-FA group experienced robust increases in both strength and power. The data provided in the present study exhibit strong evidence that HMB-FA interacts with the training stimulus itself. While our design was certainly innovative, the outcomes found agreed with the past literature. For example, HMB has been demonstrated to attenuate decreases in power and LBM in calorically restricted judo athletes subjected to high-intensity training loads (Hunga et al. 2010). HMB supplementation has also been found to acutely blunt rises in cortisol following resistance training (Kraemer et al. 2009), as well as decrease or prevent the rise in serum indices of muscle damage (Nissen et al. 1996; van Someren et al. 2005), along with subjective measures of recovery following rigorous acute training regimens (van Someren et al. 2005).

## Conclusions

The collective findings of our current study suggest that supplementation with HMB-FA in combination with a high-intensity, frequently undulating periodization training model results in increases in LBM, muscle hypertrophy, strength, and power. Moreover, when faced with greater training frequencies, as demonstrated with the overreaching cycle of training, HMB-FA may prevent typical declines in performance that are characteristic of overreaching. Future research should seek to elucidate the underlying mechanisms of HMB-FA in minimizing the negative and improving the positive effects of high-intensity training adaptations.

## Electronic supplementary material

Below is the link to the electronic supplementary material.

Supplementary material 1 (DOCX 42 kb)

## Abbreviations

HMB: beta-Hydroxy-beta-methylbutyrate
HMB-Ca: Calcium HMB
CK: Creatine kinase
HMB-FA: HMB free acid
LBM: Lean body mass
1-RM: One-repetition maximum
VL: Vastus lateralis
VI: Vastus intermedius
