# Supplementary material for: The effects of 12 weeks of beta-hydroxy-beta-methylbutyrate free acid supplementation on muscle mass, strength, and power in resistance-trained individuals: a randomized, double-blind, placebo-controlled study
Source: Eur J Appl Physiol. 2014 Mar 6;114(6):1217–27. doi: 10.1007/s00421-014-2854-5 (PMC4019830; doi:10.1007/s00421-014-2854-5)
Supplement: Supplementary file 1 — Supplementary material 1 (DOCX 42 kb) [file 421_2014_2854_MOESM1_ESM.docx]

**Table S1.** Phase 1 of the training cycle (Daily Undulating Periodization).

| **Monday** | **Wednesday** | **Friday** |
| --- | --- | --- |
| Squat | Squat | Squat (5 sets) |
| Barbell Bench Press | Bench | Barbell Bench Press (5 sets) |
| Deadlifts | Deadlift | Deadlifts |
| Pull Ups/ Dips (Superset) |  | Pull Ups/Dumbbell Shoulder Press (Superset) |
| Bent Over Row |  | Bent Over Row |
| Dumbbell Shoulder Press |  | Dumbbell Shoulder Press |
| Barbell Curl/ Triceps Extension (Superset) |  | Barbell Curls/ Triceps Extension (Superset) |
| **Repetition, Set Schema** | **Repetition, Set Schema** | **Repetition, Set Schema** |
| 3 sets | 5 sets | 3 sets *(Squat and Bench= 5 sets) |
| 8-12 RM loads | 5 maximal intended velocity repetitions | 3-5 RM loads |
| 60 seconds timed rest | 180 seconds timed rest | 240 seconds timed rest |

**Table S2.** Phase 2 of the training cycle (Overreaching).

| **Monday** | **Tuesday** | **Wednesday** | **Thursday** | **Friday** | **Saturday** |
| --- | --- | --- | --- | --- | --- |
| Squat | Leg Press | Squat | Leg Press | Squat 1 RM | Wingate and Maximal Power Testing |
| Barbell Bench Press | Barbell Bench Press | Barbell Bench Press | Barbell Bench Press | Bench Press 1 RM |  |
| Deadlifts | Military Press | Deadlifts | Military Press | Deadlift 1 RM |  |
| Pull Ups/ Dips (Superset) | Supinated Pull Ups/ Dips (Superset) | Pull Ups/ Dips (Superset) | Supinated Pull Ups/ Dips (Superset) |  |  |
| Bent Over Row | Bent Over Row | Bent Over Row | Bent Over Row |  |  |
| Dumbbell Shoulder Press | Hammer Curls/ Close Grip Bench (Superset) | Dumbbell Shoulder Press | Hammer Curls/ Close Grip Bench (Superset) |  |  |
| Barbell Curl/ Triceps Extension (Superset) |  | Barbell Curl/ Triceps Extension (Superset) |  |  |  |
| **Repetition, Set Schema** | **Repetition, Set Schema** | **Repetition, Set Schema** | **Repetition, Set Schema** | **Repetition, Set Schema** |  |
| 3 sets | 3 sets | 3 sets | 3 sets | 3 Maximal Attempts (Highest Counted as 1RM) |  |
| 8 RM loads | 8 RM loads | 12 RM loads | 12 RM loads | 1 RM load |  |
| 60 seconds timed rest | 60 seconds timed rest | 60 seconds timed rest | 60 seconds timed rest | 5 minutes timed rest |  |
| 75% 1 RM | 75% 1 RM | 65% 1 RM | 65% 1 RM |  |  |

**Table S3.** Phase 3 of the training cycle (Taper).

| **Week 11** | | | **Week 12** | | |
| --- | --- | --- | --- | --- | --- |
| **Monday** | **Wednesday** | **Friday** | **Monday** | **Wednesday** | **Friday** |
| Squat | Squat (3 sets) | Squat | Squat (3 sets) | Squat | Squat 1 RM |
| Barbell Bench Press | Barbell Bench Press  (3 sets) | Barbell Bench Press | Barbell Bench Press  (3 sets) | Barbell Bench Press | Bench Press 1 RM |
| Deadlifts | Deadlifts | Deadlifts | Deadlifts | Deadlifts | Deadlift 1 RM |
|  | Pull Ups/Dumbbell Shoulder Press (Superset) |  | Pull Ups/Dumbbell Shoulder Press (Superset) |  |  |
|  | Bent Over Row |  | Bent Over Row |  |  |
|  | Dumbbell Shoulder Press |  | Dumbbell Shoulder Press |  |  |
|  | Barbell Curls/ Triceps Extension (Superset) |  | Barbell Curls/ Triceps Extension (Superset) |  |  |
| **Repetition, Set Schema** | **Repetition, Set Schema** | **Repetition, Set Schema** | **Repetition, Set Schema** | **Repetition, Set Schema** | **Repetition, Set Schema** |
| 5 sets | 1 set *(Squat and Bench= 3 sets) | 5 sets | 1 set *(Squat and Bench= 3 sets) | 5 sets | 3 Maximal Attempts (Highest Counted as 1RM) |
| 5 maximal intended velocity repetitions | 3-5 RM loads | 5 maximal intended velocity repetitions | 3-5 RM loads | 5 maximal intended velocity repetitions | 1 RM load |
| 180 seconds timed rest | 240 seconds timed rest | 180 seconds timed rest | 240 seconds timed rest | 180 seconds timed rest | 5 minutes timed rest |
| 40-60% 1RM | > 90 % 1 RM | 40-60% 1RM | > 90 % 1 RM | 40-60% 1RM |  |

**Table S4. Blood chemistry values in males undergoing a 12-week periodized resistance training protocol and supplemented with HMB Free Acid.^*^**

|  | Placebo | | | | |  | HMB Free Acid | | | | |  |
| --- | --- | --- | --- | --- | --- | --- | --- | --- | --- | --- | --- | --- |
|  | Before | SEM^†^ | After | SEM | % Change |  | Before | SEM | After | SEM | % Change | *P* Value^‡^ |
| Glucose, mmol/l | 4.51 | 0.09 | 4.51 | 0.10 | 0.1 |  | 4.51 | 0.08 | 4.64 | 0.05 | 2.9 | 0.44 |
| Blood Urea Nitrogen, mmol/l | 5.59 | 0.29 | 5.79 | 0.34 | 3.5 |  | 5.91 | 0.22 | 6.26 | 0.28 | 6.0 | 0.85 |
| Creatinine, μmol/l | 86.6 | 2.6 | 85.5 | 2.2 | -1.4 |  | 87.8 | 2.9 | 90.5 | 4.6 | 3.0 | 0.62 |
| eGRF, ml/min | 102 | 1.6 | 104 | 2.2 | 2.0 |  | 103 | 4.4 | 104 | 4.6 | 1.1 | 0.92 |
| Sodium, mmol/l | 140 | 0.7 | 140 | 0.4 | 0.1 |  | 140 | 0.3 | 140 | 0.4 | 0.6 | 0.33 |
| Potassium, mmol/l | 4.3 | 0.1 | 4.3 | 0.1 | -0.8 |  | 4.3 | 0.1 | 4.1 | 0.1 | -3.2 | 0.82 |
| Chloride, mmol/l | 101 | 0.4 | 101 | 0.5 | 0.1 |  | 101 | 0.5 | 101 | 0.3 | 0.7 | 0.44 |
| CO_2_, mmol/l | 24.7 | 0.7 | 25.2 | 0.4 | 2.3 |  | 23.5 | 0.4 | 24.5 | 0.3 | 4.3 | 0.75 |
| Calcium, mmol/l | 2.40 | 0.03 | 2.39 | 0.03 | -0.5 |  | 2.44 | 0.02 | 2.41 | 0.02 | -1.3 | 0.63 |
| Protein, g/l | 71.2 | 0.9 | 71.6 | 1.6 | 0.5 |  | 72.7 | 1.3 | 72.4 | 0.9 | -0.5 | 0.26 |
| Albumin, g/l | 46.6 | 0.6 | 45.8 | 0.9 | -1.7 |  | 47.0 | 0.4 | 45.6 | 0.6 | -2.9 | 0.11 |
| Globulin, g/l | 25.7 | 0.5 | 25.8 | 0.6 | 0.4 |  | 24.7 | 0.7 | 25.4 | 0.7 | 2.6 | 0.41 |
| A:G Ratio | 1.8 | 0.1 | 1.8 | 0.1 | -2.0 |  | 1.9 | 0.1 | 1.8 | 0.1 | -5.4 | 0.10 |
| Total Bilirubin, μmol/l | 11.8 | 2.2 | 11.0 | 2.1 | -6.5 |  | 11.3 | 1.7 | 10.9 | 1.9 | -4.1 | 0.85 |
| Alkaline Phosphatase, IU/l | 76.6 | 3.5 | 80.8 | 3.8 | 5.5 |  | 93.1 | 6.9 | 100.5 | 6.4 | 7.9 | 0.93 |
| Aspartate Aminotransferase, IU/l | 23.9 | 1.8 | 22.6 | 1.5 | -5.6 |  | 27.5 | 1.5 | 26.7 | 1.9 | -2.6 | 0.97 |
| Alanine Aminotransferase, IU/l | 27.6 | 2.9 | 27.4 | 3.0 | -0.4 |  | 23.2 | 1.7 | 23.2 | 1.5 | 0.0 | 0.82 |

^*^Values before and after 12 weeks of either placebo (n=9) or HMB Free Acid (3 g/d, n=11) administration.

^†^Standard error of the mean.

^‡^*P* value for treatment*time effect, the mixed model included data from weeks 4 and 8 (not shown).

**Table S5. Blood hematology** **values in males undergoing a 12-week periodized resistance training protocol and supplemented with HMB Free Acid.^*^**

|  | Placebo | | | | |  | HMB Free Acid | | | | |  |
| --- | --- | --- | --- | --- | --- | --- | --- | --- | --- | --- | --- | --- |
|  | Before | SEM^†^ | After | SEM | % Change |  | Before | SEM | After | SEM | % Change | *P* Value^‡^ |
| WBC, x10^9^/l | 6.0 | 0.4 | 6.0 | 0.4 | 0.4 |  | 5.7 | 0.3 | 5.8 | 0.3 | 1.6 | 0.62 |
| RBC, x10^12^/l | 5.2 | 0.1 | 5.2 | 0.1 | 0.2 |  | 5.1 | 0.1 | 5.0 | 0.1 | -1.1 | 0.41 |
| Hemoglobin, g/l | 156 | 3.1 | 154 | 2.5 | -1.3 |  | 156 | 2.6 | 154 | 1.7 | -0.9 | 0.76 |
| Hematocrit, l/l | 0.46 | 0.01 | 0.46 | 0.01 | -0.5 |  | 0.46 | 0.01 | 0.46 | 0.01 | -1.1 | 0.55 |
| MCV, μm^3^ | 89.1 | 0.6 | 88.7 | 0.9 | -0.5 |  | 91.4 | 1.0 | 90.3 | 0.8 | -1.2 | 0.59 |
| MCH, pg/cell | 30.2 | 0.3 | 29.9 | 0.3 | -0.8 |  | 30.6 | 0.4 | 30.1 | 0.7 | -1.8 | 0.98 |
| MCHC, g/l | 338 | 3.2 | 335 | 2.6 | -0.8 |  | 335 | 3.2 | 334 | 8.7 | -0.3 | 0.92 |
| RDW, % | 13.4 | 0.1 | 13.4 | 0.1 | 0.4 |  | 13.3 | 0.1 | 13.2 | 0.1 | -0.3 | 0.25 |
| Platelets, x10^9^/l | 250 | 9.3 | 240 | 9.3 | -4.0 |  | 230 | 8.7 | 228 | 7.0 | -1.0 | 0.90 |
| Neutrophils, % | 46.3 | 3.5 | 47.3 | 2.8 | 2.2 |  | 44.2 | 3.5 | 45.1 | 2.0 | 2.1 | 0.83 |
| Lymphocytes, % | 39.7 | 3.6 | 38.2 | 2.9 | -3.6 |  | 43.3 | 3.3 | 42.4 | 1.9 | -2.1 | 0.72 |
| Monocytes, % | 10.1 | 0.7 | 9.3 | 0.7 | -7.7 |  | 10.2 | 0.9 | 9.2 | 0.4 | -9.8 | 0.90 |
| Eosinophils, % | 3.8 | 0.5 | 3.8 | 0.5 | 0.0 |  | 3.2 | 0.6 | 3.2 | 0.6 | 0.0 | 1.00 |
| Basophils, % | 0.7 | 0.2 | 0.8 | 0.1 | 16.7 |  | 0.3 | 0.1 | 0.4 | 0.2 | 33.3 | 0.67 |
| Neutrophils, x10^9^/l | 3.2 | 0.6 | 3.3 | 0.5 | 2.4 |  | 2.7 | 0.3 | 2.9 | 0.2 | 8.8 | 0.46 |
| Lymphocytes, x10^9^/l | 2.4 | 0.2 | 2.5 | 0.3 | 5.0 |  | 2.5 | 0.2 | 2.7 | 0.1 | 6.8 | 0.89 |
| Monocytes, x10^9^/l | 0.67 | 0.09 | 0.62 | 0.09 | -6.7 |  | 0.60 | 0.04 | 0.58 | 0.03 | -3.0 | 0.93 |
| Eosinophils, x10^9^/l | 0.22 | 0.02 | 0.22 | 0.02 | 0.0 |  | 0.18 | 0.03 | 0.20 | 0.02 | 10.0 | 0.35 |
| Basophils, x10^9^/l | 0.02 | 0.01 | 0.01 | 0.01 | -50.0 |  | 0.01 | 0.01 | 0.0 | 0.0 | -100.0 | 0.42 |

^*^Values before and after 12 weeks of either placebo (n=9) or HMB Free Acid (3 g/d, n=11) administration.

^†^Standard error of the mean.

^‡^*P* value for treatment*time effect, the mixed model included data from weeks 4 and 8 (not shown).

**Table S6. Urinalysis** **values in males undergoing a 12-week periodized resistance training protocol and supplemented with HMB Free Acid.^*^**

|  | Placebo | | | | |  | HMB Free Acid | | | | |  |
| --- | --- | --- | --- | --- | --- | --- | --- | --- | --- | --- | --- | --- |
|  | Before | SEM^†^ | After | SEM | % Change |  | Before | SEM | After | SEM | % Change | *P* Value^‡^ |
| Urine Specific Gravity | 1.020 | 0.002 | 1.021 | 0.003 | 0.02 |  | 1.023 | 0.001 | 1.026 | 0.001 | 0.3 | 0.29 |
| Urine pH | 6.56 | 0.15 | 6.44 | 0.13 | -1.7 |  | 6.14 | 0.10 | 6.18 | 0.10 | 0.7 | 0.43 |
| Urine Urobilinogen, Semi-Quantative, EU/dl | 0.189 | 0.011 | 0.211 | 0.011 | 11.8 |  | 0.209 | 0.009 | 0.191 | 0.009 | -8.7 | 0.18 |

^*^Values before and after 12 weeks of either placebo (n=9) or HMB Free Acid (3 g/d, n=11) administration. Additional values were measured

and reported qualitatively: Color: all reported as yellow; Appearance: all reported as clear; WBC Esterase, Protein, Glucose, Ketones, Occult Blood,

Bilirubin, and Nitrite: all reported as negative.

^†^Standard error of the mean.

^‡^*P* value for treatment*time effect, the mixed model included data from weeks 4 and 8 (not shown).
